# Dexmedetomidine as a potential neuroprotective agent against Alzheimer’s disease: molecular mechanisms and therapeutic perspectives

**DOI:** 10.3389/fnagi.2026.1952923

**Published:** 2026-10-02

**Authors:** Jun Kang, Yongbo Duan, Jiaxuan Bi, Jiangping Liu, Wenjun Yan

**Affiliations:** 1 First Clinical Medical College of Lanzhou University, Lanzhou, China; 2 Department of Anesthesiology, Gansu Provincial Hospital, Lanzhou, China; 3 Institute of Clinical Research and Translational Medicine, Gansu Provincial Hospital, Lanzhou, China

**Keywords:** Alzheimer’s disease, amyloid-β, dexmedetomidine, neuroinflammation, neuroprotection

## Abstract

Alzheimer’s disease (AD) is a neurodegenerative disorder characterized by extracellular deposition of amyloid-β (Aβ), intracellular hyperphosphorylation of microtubule-associated protein tau and chronic neuroinflammation. At present, effective disease-modifying therapies for AD remain limited. Dexmedetomidine (Dex), a highly selective α_2_-adrenergic receptor agonist, has been reported in recent years to exert significant neuroprotective effects. Accumulating evidence indicates that Dex may ameliorate AD pathology through multiple mechanisms. In terms of Aβ metabolism, Dex reduces Aβ production by downregulating β-site amyloid precursor protein-cleaving enzyme 1 (BACE1) expression. With respect to tau pathology, Dex may counteract abnormal tau hyperphosphorylation in AD-related models, although relatively high experimental doses have also been associated with increased tau phosphorylation. Regarding immune regulation, Dex attenuates neuroinflammatory signaling by inhibiting NOD-like receptor family pyrin domain-containing 3 (NLRP3) inflammasome activation and related pro-inflammatory pathways. In addition, Dex modulates neurotrophic support, ferroptosis, autophagic flux, and synaptic homeostasis. These multitarget effects have been observed across heterogeneous preclinical models and dose ranges; however, an AD-specific therapeutic window and long-term safety profile remain to be established. This narrative review synthesizes the available preclinical evidence on the multitarget actions of Dex in AD-related models and critically evaluates dose dependence, pharmacokinetic and safety considerations, and current translational limitations.

## 1 Introduction

Alzheimer’s disease (AD) is the most common form of dementia, accounting for 60%–80% of all dementia cases (Rostagno, 2023). It is estimated that more than 50 million individuals worldwide are currently living with dementia (Gustavsson et al., 2023). Recent epidemiological projections suggest that by 2050, the prevalence of dementia in Europe will double, while the global dementia burden will increase to approximately three times the current level (Scheltens et al., 2021). AD is clinically characterized by progressive memory impairment and cognitive decline (Alzheimer’s Association, 2012). Its hallmark pathological features include extracellular deposition of amyloid-β (Aβ) to form senile plaques and intracellular aggregation of abnormally phosphorylated microtubule-associated protein tau to form neurofibrillary tangles (NFTs) (Lane et al., 2018). Although anti-amyloid monoclonal antibodies, including lecanemab and donanemab, have introduced disease-modifying treatment options for selected patients with early AD, their clinical benefits remain limited and are accompanied by important safety, monitoring, and eligibility considerations (Van Dyck et al., 2023; Sims et al., 2023). Therefore, there remains a substantial need for additional therapeutic strategies targeting the multifactorial pathophysiology of AD (Rostagno, 2023). With the continued acceleration of population aging in China, AD has become a major public health issue affecting the health and quality of life of older adults (Jia et al., 2020). Therefore, identifying safe and effective interventional strategies for AD is of considerable social and clinical importance.

Dexmedetomidine (Dex) is a highly selective α_2_-adrenergic receptor (α₂-AR) agonist with sedative, anxiolytic, and analgesic properties (Weerink et al., 2017). In recent years, multiple studies have demonstrated that Dex exerts neuroprotective effects by suppressing inflammatory responses (Sun et al., 2019; Li et al., 2020; Li et al., 2018). Mechanistically, Dex activates α₂-ARs and downregulates key inflammatory mediators, including interleukin-1β and nuclear factor kappa B (NF-κB), thereby attenuating neuroinflammation (Li et al., 2020). In experimental models, Dex has been reported to attenuate neuroinflammation and cognitive impairment, supporting further investigation of its potential relevance to AD (Li et al., 2020; Luo et al., 2022; Ma et al., 2024; Li et al., 2025).

However, the multiple mechanisms through which Dex may influence AD pathology have not yet been comprehensively integrated. Therefore, this narrative review summarizes the available preclinical and translational evidence regarding the potential neuroprotective effects of Dex in AD, with particular emphasis on its regulation of Aβ accumulation, tau pathology, neuroinflammation, oxidative stress, mitochondrial dysfunction, autophagic impairment, neuronal death, and synaptic dysfunction.

## 2 Methods

### 2.1 Literature search strategy

A structured literature search was conducted to identify studies relevant to the potential neuroprotective effects of dexmedetomidine (Dex) in Alzheimer’s disease (AD). PubMed and Web of Science were searched from database inception through September 2, 2026. The search used the terms “dexmedetomidine” AND (“Alzheimer’s disease” OR “Alzheimer disease”) to identify experimental and translational studies examining the potential effects and mechanisms of Dex in the context of AD.

Additional relevant mechanistic, pharmacological, pharmacokinetic, and safety studies were identified through targeted supplementary searches and screening of the reference lists of relevant articles. The search was designed primarily to identify evidence directly examining Dex in AD or AD-related experimental models. Additional mechanistic, pharmacological, pharmacokinetic, and safety studies conducted outside AD-specific models were considered when they provided information directly relevant to the interpretation of the biological plausibility, dose dependence, pharmacological characteristics, or potential limitations of Dex in AD.

### 2.2 Study eligibility and selection

Studies were considered eligible if they investigated Dex in AD-specific or AD-related experimental models and reported findings relevant to AD pathology, neuroprotection, cognitive or behavioral outcomes, or molecular mechanisms pertinent to this review. Both *in vivo* and *in vitro* studies were considered. Studies conducted in non-AD experimental settings were included selectively when they provided mechanistic, pharmacological, pharmacokinetic, or safety evidence directly relevant to the interpretation of Dex in AD.

Records were excluded if they did not investigate Dex or did not provide evidence relevant to AD-related pathological mechanisms, neuroprotection, pharmacological properties, or safety considerations. Studies whose findings could not be meaningfully related to the mechanistic or translational scope of this review were also excluded.

Titles and abstracts of potentially relevant records were independently screened by Jun Kang and Yongbo Duan. Potentially eligible studies subsequently underwent independent full-text assessment by the same two reviewers. Disagreements regarding study eligibility were resolved through discussion and, when necessary, adjudication by Wenjun Yan.

### 2.3 Evidence synthesis

This review was conducted as a structured narrative review rather than a systematic review or meta-analysis. Because the available evidence differed substantially with respect to experimental models, Dex doses or concentrations, routes of administration, treatment durations, and outcome measures, quantitative pooling was considered inappropriate. The eligible evidence was therefore synthesized narratively and organized according to major pathological and mechanistic domains relevant to AD, including Aβ pathology, tau phosphorylation, neuroinflammation, neuronal apoptosis and ferroptosis, oxidative stress, mitochondrial dysfunction, autophagic flux, and synaptic function.

Evidence from AD-specific models was given primary emphasis, whereas findings from non-AD experimental settings were used primarily to provide mechanistic, pharmacological, pharmacokinetic, or safety context and were not treated as direct evidence of therapeutic efficacy in AD. Dose-dependent effects, pharmacokinetic characteristics, and potential safety concerns were additionally considered when evaluating the translational relevance of the available evidence. Given the narrative design of the review, no quantitative meta-analysis or formal risk-of-bias assessment was performed.

## 3 Multitarget mechanisms by which Dex ameliorates AD pathology

Given the substantial heterogeneity in experimental models, administration routes, treatment durations, and Dex exposures, the available preclinical evidence does not support a single universally optimal dose for AD. Representative *in vivo* and *in vitro* studies are summarized in Table 1, including the experimental models, Dex doses or concentrations, treatment schedules, and major outcomes.

**Table 1:** Representative preclinical studies evaluating dexmedetomidine in Alzheimer’s disease-related and AD-relevant safety models, with experimental doses/concentrations and major outcomes.

Study	Experimental model	Dex dose/concentration	Route and treatment schedule	Main findings/proposed mechanism
Sun et al. (2020)	Aβ1–42-induced AD mouse model; hippocampal neuronal apoptosis and cognitive impairment	NR (not reported in full-text manuscript)	Tail-vein injection; exact dose, frequency and duration are not described in the original paper	Dex increased miR-129 expression and reduced hippocampal neuronal apoptosis and cognitive impairment through the miR-129/YAP1/JAG1 axis
Guo et al. (2021)	Aβ1–42-treated SH-SY5Y and PC12 cells	1 μM	Dex for 24 h, followed by 300 nM Aβ1–42 for another 24 h	Reduced Aβ-induced apoptosis and ROS; increased antioxidant capacity; miR-151-3p enhanced Dex neuroprotection by targeting DAPK1/TP53
Hu et al. (2022)	APP/PS1 transgenic mice	20 μg/kg	Intraperitoneal injection, once daily for 4 consecutive days; behavioral assessment performed 2 weeks after the final Dex injection	Improved spatial memory; reduced hippocampal neuronal apoptosis, inflammatory factors and oxidative stress; ↑miR-214-5p, ↓SUZ12
Luo et al. (2022)	5 × FAD mice	100 μg/kg	Intragastric administration, once daily for 14 days	Improved cognition; reduced hippocampal Aβ deposition, BACE1, IL-1β and NF-κB signaling; effects were α₂-AR dependent
Qiao et al. (2023)	Aβ-induced C57BL/6 mouse AD model; complementary neuronal-cell experiments	*In vivo*: 20 μg/kg/day; *in vitro*: 5 μM	Mouse: i.p., once daily for 4 weeks; cellular experiments evaluated Aβ-induced injury	Improved learning and memory and reduced ferroptotic injury; activated mTOR and increased SLC7A11/GPX4 while reducing iron-associated injury
Tan et al. (2023)	SH-SY5Y cells, AD-like injury induced by Aβ1–42	10 μM	Co-treatment with Dex after 24 h of Aβ₁₋₄₂ exposure; no in vivo animal experiments	Dex ↓lncRNA SNHG14, ↑HSPB8 through regulation of UPF1, reduced apoptosis and p-Tau
Lee et al. (2023)	C57BL/6 N mice receiving i.c.v. Aβ25–35; organotypic hippocampal slice cultures (OHSCs)	*In vivo*: 40 or 80 μg/kg; OHSC: 0.5, 1, 2.5, 5 μM	Mouse: i.p. twice at 30-min intervals; OHSC: Aβ25–35 + Dex	Dex reduced p-Tau, improved PSD-95/p-CaMKII balance and restored autophagic flux. In OHSCs, ≥1 μM produced similarly strong protection
Wang et al. (2019)	Primary hippocampal neurons and cortical astrocytes exposed to 30 nM Aβ	0.1, 1, 10 μM	In vitro co-treatment	Dex improved cell viability and reduced apoptosis by restoring BDNF production through HDAC2/HDAC5-associated regulation
Lian et al. (2024)	Aβ-injected mouse AD model	20 μg/kg	Intraperitoneal injection, once daily for 4 consecutive days, initiated on day 21 post-Aβ injection; behavioral tests performed ~2 weeks after dosing	Reduced hippocampal neuroinflammation by ↑miR-204-3p and ↓FBXL7; inhibition of miR-204-3p or FBXL7 overexpression reversed the effect
Ma et al. (2024)	Male C57BL/6 J mice with i.c.v. Aβ-induced AD-like pathology	25 and 50 μg/kg	i.p.; initial dose comparison for 8 days; subsequent mechanistic experiments used 25 μg/kg for 7 days	Both doses improved cognitive and anxiety-like behavior and reduced neuronal injury/CXCL2-related inflammation
Li et al. (2025)	Male C57BL/6 J mice with i.c.v. Aβ1–42	25 or 50 μg/kg/day	i.p., once daily for 7 consecutive days, beginning 7 days after Aβ injection	Improved memory and anxiety-like behavior; preserved neurons; modulated neurogenesis, CXCL2/inflammation and oxidative stress
Yang et al. (2025)	APP/PS1 mice + AD bioinformatics + molecular docking	20 μg/kg	Single intraperitoneal injection; mice were sacrificed 2 h post-injection for tissue collection	Dex restored hippocampal RGS7 expression and reduced Aβ plaque burden; docking predicted a Dex–RGS7 interaction; RGS7 was linked to immune-cell profiles
Nazli et al. (2026)	Adult zebrafish with cerebroventricular Aβ42; larval zebrafish toxicity experiments	Adult: 2 μL of 250 μM Dex; larvae: 250 and 500 μM	Adult: retro-orbital injection after Aβ model establishment; larval exposure: 24–48 h	Reduced Aβ accumulation, amyloidogenic gene expression, glial activation and inflammatory responses; improved behavioral abnormalities
Wu et al. (2026)	5 × FAD mice; Aβ1–42-treated SH-SY5Y cells and primary neurons	*In vivo*: 4 μg/kg/day; in vitro: 2.5, 5, 10 μM	Mouse: i.p. once daily for 4 weeks; cells: Aβ1–42 5 μM for 24 h followed by Dex for 1 h	Improved spatial learning/memory and reduced Aβ plaques; ↓ROS, ↑XIAP, ↓MDM2/p53/cleaved-caspase-3; effects were α₂-AR/XIAP dependent
Whittington et al. (2015)‡	C57BL/6 mice, hTau transgenic mice, and human tau-expressing SH-SY5Y cells; AD-relevant tau safety model	*In vivo*: 300 μg/kg; in vitro Dex tested across increasing concentrations	Mouse: single i.p. administration; acute tau assessment; cell experiments assessed dose−/time-dependent tau phosphorylation	Dex increased AT8, CP13 and PHF-1 tau phosphorylation, promoted tau aggregation in hTau mice and impaired spatial memory; effect was α₂-AR dependent

Aβ, amyloid-β; AD, Alzheimer’s disease; APP/PS1, amyloid precursor protein/presenilin 1; BACE1, β-site amyloid precursor protein-cleaving enzyme 1; BDNF, brain-derived neurotrophic factor; Dex, dexmedetomidine; i.c.v., intracerebroventricular; i.p., intraperitoneal; OHSCs, organotypic hippocampal slice cultures; ROS, reactive oxygen species; 5 × FAD, five familial Alzheimer’s disease mutations.

The doses and concentrations shown represent experimentally tested effective or adverse exposure levels rather than an established optimal therapeutic dose. Direct comparisons across studies should be interpreted cautiously because of substantial heterogeneity in species, AD model, administration route, exposure duration, and outcome measures. ‡Whittington et al. (2015) used tau-related models and is included as AD-relevant dose-safety evidence rather than as an AD-specific therapeutic model.

### 3.1 Regulation of tau phosphorylation

Tau is a key microtubule-associated protein predominantly distributed in neuronal axons, where it maintains microtubule structural stability and promotes axonal transport through dynamic binding to microtubules (Hong et al., 2025). Under pathological conditions associated with AD, tau undergoes conformational changes due to abnormal post-translational modifications, including hyperphosphorylation and acetylation, leading to its dissociation from microtubules and aggregation into soluble oligomers or insoluble NFTs (Chinnathambi, 2025). These pathological tau species not only disrupt microtubule networks and mitochondrial function, thereby inducing synaptic dysfunction, but also spread to adjacent brain regions through transneuronal propagation. The pattern of tau deposition is positively correlated with the severity of cognitive impairment in patients with AD (Vogel et al., 2021). Tau phosphorylation is generally governed by a dynamic balance between kinases, including glycogen synthase kinase-3β (GSK-3β), mitogen-activated protein kinase/extracellular signal-regulated kinase (MAPK/ERK), and calcium/calmodulin-dependent protein kinase II (CaMKII), as well as phosphatases such as protein phosphatase 2A (PP2A) (Islam et al., 2025; Zhang et al., 2020).

Current evidence regarding the effects of Dex on tau pathology suggests a bidirectional regulatory profile. On the one hand, Dex may induce tau phosphorylation under specific conditions. Intraperitoneal administration of Dex at 300 μg/kg in C57BL/6 mice significantly increased tau phosphorylation at AT8, CP13, and PHF-1 epitopes in the hippocampus. This effect was α₂-AR-dependent and dose-dependent, but independent of the activation of classical kinases such as GSK-3β. These findings raise a preclinical safety concern regarding the dose-dependent effects of Dex on tau phosphorylation, although their clinical relevance remains uncertain (Whittington et al., 2015; Whittington et al., 2013). In contrast, AD-related studies have also reported attenuation of tau-associated abnormalities under model-specific experimental conditions (Tan et al., 2023; Lee et al., 2023). However, substantial heterogeneity in experimental dose, route of administration, and exposure duration currently limits direct comparison across studies, and an AD-specific therapeutic window for Dex has not yet been established (Luo et al., 2022; Li et al., 2025; Lee et al., 2023; Whittington et al., 2015). Mechanistically, Dex suppresses the long non-coding RNA (lncRNA) small nucleolar RNA host gene 14 (SNHG14)/UPF1 RNA helicase (UPF1) signaling axis and upregulates heat shock protein family B member 8 (HSPB8) expression, thereby antagonizing Aβ1–42-induced tau hyperphosphorylation (Tan et al., 2023). Lee et al. reported that, in both mice and organotypic hippocampal slice cultures, Dex reduced Aβ25–35-induced p-Tau levels and ameliorated associated hippocampal and synaptic abnormalities (Lee et al., 2023). Overall, Dex may attenuate AD-related tau pathology by modulating proteostasis and associated pathological cascades. However, the precise dose threshold and optimal timing of administration require further clarification. Notably, the bidirectional effects of Dex on tau regulation suggest that its actions are highly dose-dependent and model-dependent. Future studies should systematically evaluate its safety window across different pathological stages of AD and explore the selective coupling mechanisms between Dex and downstream α₂-AR signaling pathways to enable precision intervention.

Taken together, the available evidence does not define a universal optimal dose of Dex. Rather, its effects appear to vary according to experimental model, administration route, and exposure level, with relatively high exposure associated with increased tau phosphorylation under certain conditions.

### 3.2 Inhibition of aβ deposition

As a highly selective α₂-AR agonist, Dex has shown considerable neuroprotective potential and regulatory effects on amyloid pathology in AD. In retinal neurodegeneration-related *in vitro* models, Dex at concentrations as low as 0.01 μM improved cell viability and reduced Aβ immunoreactivity (Nizari et al., 2016). Together with findings obtained using other α₂-AR agonists such as brimonidine, these observations suggest that α₂-adrenergic signaling may influence amyloid precursor protein (APP) processing. However, direct evidence that Dex shifts APP processing toward the non-amyloidogenic pathway in AD-specific models remains limited (Nizari et al., 2016). Dex has also shown anti-amyloidogenic effects *in vivo*. Through an α₂-AR-dependent pathway, Dex significantly suppresses Aβ plaque deposition in the hippocampus, particularly in the dentate gyrus, of five familial Alzheimer’s disease mutations (5 × FAD) mice (Luo et al., 2022). Luo et al. demonstrated that Dex effectively downregulates the transcriptional and protein expression levels of BACE1, thereby reducing Aβ generation and decreasing both plaque area and mean optical density. The α₂-AR antagonist yohimbine reversed these effects, further supporting the involvement of α₂-AR signaling (Luo et al., 2022). Notably, a recent study integrating bioinformatics, molecular docking, and *in vivo* validation identified RGS7 as a potential Dex-related target in AD (Yang et al., 2025). Bioinformatic analyses showed reduced RGS7 expression in AD-related datasets (Yang et al., 2025). Molecular docking predicted a potential interaction between Dex and RGS7 (Yang et al., 2025), while subsequent *in vivo* analyses showed that Dex treatment was associated with increased hippocampal RGS7 expression and reduced Aβ deposition in APP/PS1 mice (Yang et al., 2025).

In addition, Dex has been reported to significantly reduce brain Aβ deposition and downregulate multiple amyloidogenic genes in an Aβ42-induced adult zebrafish model of AD, thereby suppressing the amyloid cascade at the transcriptional level (Nazli et al., 2026). Collectively, Dex may attenuate amyloid pathology in AD by modulating multiple steps of APP processing and Aβ generation. However, most available evidence focuses on the regulation of Aβ production. Whether Dex affects Aβ clearance pathways, including microglia-mediated phagocytic clearance and enzymatic degradation systems in the brain, remains insufficiently explored. Future studies should integrate emerging research areas, such as glymphatic system function, to further clarify these mechanisms.

### 3.3 Attenuation of chronic neuroinflammation

Inhibition of neuroinflammation is one of the central mechanisms by which Dex exerts neuroprotective effects. The NLRP3 inflammasome is a key effector complex of the innate immune system, consisting of the NLRP3 pattern-recognition receptor, the adaptor protein apoptosis-associated speck-like protein containing a caspase recruitment domain, and the effector protease caspase-1 (Xu et al., 2025). In AD, Aβ- and tau-related danger signals can promote NLRP3 inflammasome activation and caspase-1-dependent maturation of IL-1β and IL-18, thereby amplifying neuroinflammation and contributing to neuronal and synaptic dysfunction (Xu et al., 2025; Guo et al., 2026; Liang et al., 2022). Dex may modulate neuroinflammatory signaling through several mechanisms. In LPS/ATP-stimulated microglia, Dex was reported to inhibit the c-Fos/NLRP3/caspase-1 signaling cascade and c-Fos-mediated transcriptional activation of NLRP3, thereby reducing inflammasome activation and inflammatory mediator release (Li et al., 2018). On the other hand, Dex exerts broad immunomodulatory activity through α₂-AR-dependent pathways. Dex inhibits NF-κB p65 activation in inflammatory and AD-related models (Li et al., 2020; Luo et al., 2022) and downregulates CXCL2 in Aβ-induced AD models (Ma et al., 2024; Li et al., 2025). These effects may contribute to attenuation of downstream neuroinflammatory signaling.

Dex also modulates neuroinflammatory responses through ncRNA-related regulatory mechanisms. At the level of non-coding RNA (ncRNA) regulation, Dex modulates microRNA-mediated signaling axes, including microRNA-214-5p (miR-214-5p)/suppressor of zeste 12 homolog (SUZ12) and microRNA-204-3p (miR-204-3p)/F-box and leucine-rich repeat protein 7 (FBXL7), thereby attenuating pro-inflammatory responses in AD-related models and contributing to the restoration of neuroimmune homeostasis (Hu et al., 2022; Lian et al., 2024). Bioinformatic analyses further associated RGS7 expression with altered immune-cell infiltration profiles in AD, and RGS7 was detected in microglia (Yang et al., 2025). Whether Dex directly remodels these immune-cell populations through RGS7 remains to be established. In LPS-based inflammatory injury models, Dex attenuated inflammatory cascades without markedly altering baseline inflammatory markers under control conditions (Li et al., 2020; Li et al., 2018; Chen et al., 2021), suggesting context-dependent immunomodulatory effects.

In summary, Dex may attenuate AD-related neuroinflammatory processes by modulating inflammasome activation, NF-κB/CXCL2 signaling, and ncRNA-related regulatory pathways. However, much of the mechanistic evidence derives from acute or pretreatment models, and its effects during chronic neuroinflammation, adaptive immune responses, and long-term exposure remain uncertain.

### 3.4 Inhibition of neuronal apoptosis and ferroptosis

Dex may exert neuroprotective effects by modulating neuronal apoptosis and neurotrophic signaling. Brain-derived neurotrophic factor (BDNF) plays a central role in maintaining neuronal survival, enhancing synaptic plasticity, and promoting memory formation. Reduced BDNF signaling has been reported in AD-related experimental settings (Sen et al., 2015; Hsiao et al., 2017). Notably, Aβ oligomers and apolipoprotein E4 (ApoE4) can suppress BDNF expression through HDAC-related nuclear signaling (Sen et al., 2015). Wang et al. reported that Dex effectively inhibits Aβ-induced abnormal nuclear translocation of histone deacetylases 2 and 5 (HDAC2/5), restores histone H3 acetylation, and promotes BDNF transcription and secretion. Its neurotrophic effects closely resemble those of exogenous BDNF intervention (Wang et al., 2019). In terms of anti-apoptotic mechanisms, Dex may exert anti-apoptotic effects through several ncRNA-related signaling pathways. Together, these studies implicate the miR-129/YAP1/JAG1 and miR-151-3p/DAPK1/TP53 axes in Dex-associated anti-apoptotic neuroprotection, although the regulatory direction differs between the two experimental paradigms (Sun et al., 2020; Guo et al., 2021). In addition, Dex modulates the miR-214-5p/SUZ12 axis and the lncRNA SNHG14/UPF1/HSPB8 axis, thereby contributing to neuronal survival and proteostasis (Hu et al., 2022; Tan et al., 2023). More recently, Wu et al. identified a ROS-dependent apoptotic mechanism underlying Dex-mediated neuroprotection in AD models. In Aβ1–42-exposed SH-SY5Y cells and primary neurons, Dex reduced ROS accumulation, restored X-linked inhibitor of apoptosis protein (XIAP) expression, and suppressed MDM2/p53 and cleaved-caspase-3 signaling. These protective effects were attenuated by α₂-AR blockade with yohimbine and by XIAP knockdown, supporting an α₂-AR-dependent ROS/XIAP/MDM2/p53 pathway in Dex-mediated inhibition of neuronal apoptosis (Wu et al., 2026).

Dex also protects neuronal viability by interfering with the mitochondrial intrinsic apoptotic pathway and with emerging forms of programmed cell death such as ferroptosis. In an LPS-induced hippocampal injury model, Chen et al. reported that Dex suppressed the p38 MAPK/c-Myc/chloride intracellular channel 4 (CLIC4) signaling axis, thereby preventing Bax translocation to mitochondria and cytochrome c release (Chen et al., 2021). Notably, with regard to ferroptosis, which has recently attracted considerable attention, Dex has been shown to activate the mechanistic target of rapamycin (mTOR) signaling pathway, upregulate the anti-ferroptotic regulators solute carrier family 7 member 11 (SLC7A11) and glutathione peroxidase 4 (GPX4), and reduce intracellular Fe^2+^ levels, thereby significantly alleviating Aβ-induced iron deposition and neuronal injury (Qiao et al., 2023). In Aβ-induced mouse models, Dex was also associated with reduced Aβ immunoreactivity and restoration of Bcl-2 expression, with yohimbine-sensitive effects supporting α₂-AR involvement (Ma et al., 2024; Li et al., 2025). Together, these findings suggest that Dex may engage multiple neurotrophic and anti-apoptotic pathways in AD-related models. The preliminary evidence regarding its inhibitory effect on ferroptosis expands the current understanding of Dex-mediated neuroprotection. However, evidence for Dex-mediated regulation of ferroptosis in AD remains limited to a small number of preclinical studies and should be validated using additional canonical ferroptosis markers and independent experimental models.

### 3.5 Attenuation of oxidative stress injury

Within the pathological framework of AD, Aβ deposition and the resulting oxidative stress continuously inhibit the activities of antioxidant enzymes, including superoxide dismutase (SOD) and glutathione peroxidase (GSH-Px), leading to progressive decline in total antioxidant capacity (T-AOC). This systemic failure of antioxidant defense causes excessive accumulation of reactive oxygen species (ROS), which in turn drives lipid peroxidation cascades and promotes pathological accumulation of malondialdehyde (MDA), ultimately disrupting neuronal lipid homeostasis and triggering apoptosis (Yu et al., 2020; Lin et al., 2022). Available studies suggest that Dex attenuates oxidative stress through several mechanisms (Ma et al., 2024; Hu et al., 2022; Qiao et al., 2023). On the one hand, Dex activates the mTOR signaling pathway or downregulates CXCL2 expression, thereby reducing reactive oxygen species and malondialdehyde levels while restoring the activity and content of endogenous antioxidants such as GSH-Px and SOD (Ma et al., 2024; Qiao et al., 2023). On the other hand, the antioxidant efficacy of Dex is epigenetically regulated by the miR-214-5p/SUZ12 signaling axis; overexpression of miR-214-5p further enhances its antioxidant effects (Hu et al., 2022). Nrf2/ARE signaling is an important antioxidant regulatory pathway implicated in AD-related oxidative stress (Yu et al., 2020; Lin et al., 2022). Dex has been shown to improve several oxidative-stress indices in AD-related models (Ma et al., 2024; Guo et al., 2021; Hu et al., 2022; Qiao et al., 2023). Recent evidence further showed that Dex directly reduced Aβ1–42-induced ROS accumulation in neuronal cells and that this antioxidant effect was associated with restoration of XIAP and suppression of MDM2/p53 signaling (Wu et al., 2026). However, whether these effects are mediated directly through the Nrf2/antioxidant response element (ARE) pathway remains to be established. Collectively, these findings suggest that Dex may alleviate oxidative injury in AD-related models by enhancing antioxidant defenses and limiting oxidative metabolite accumulation. However, current studies mainly focus on indirect regulation of antioxidant enzyme activity. Whether Dex directly modulates mitochondrial respiratory chain complexes or redox-sensitive transcription factors remains unclear. Future studies using high-resolution metabolomics may help define its molecular targets and regulatory hierarchy.

### 3.6 Modulation of mitochondrial dysfunction and homeostasis

Mitochondrial dysfunction is increasingly recognized as an important component of AD pathogenesis. It encompasses impaired bioenergetics, excessive ROS production, disturbed mitochondrial dynamics, and defective mitophagy, which collectively contribute to synaptic dysfunction and neuronal degeneration (Mcgill Percy et al., 2025). Direct evidence for mitochondrial protection by Dex in chronic AD models, however, remains limited. In an LPS-induced hippocampal injury model, Chen et al. reported that Dex inhibited the p38 MAPK/c-Myc/CLIC4 signaling pathway, reduced Bax-associated mitochondrial apoptotic signaling, and attenuated mitochondrial ultrastructural damage, including matrix vacuolization and disruption of mitochondrial cristae (Chen et al., 2021). The effects of Dex on mitochondrial dynamics, including fusion–fission balance and mitophagy, under chronic AD conditions remain insufficiently evaluated. Future studies combining live-cell imaging with three-dimensional electron tomography may help clarify the mechanisms underlying mitochondrial protection by Dex.

### 3.7 Restoration of impaired autophagic flux

Autophagy is a major lysosome-dependent degradative pathway that maintains neuronal proteostasis and organelle quality control, and disruption of the autophagy–lysosome system is increasingly recognized as a prominent feature of AD (Nixon and Rubinsztein, 2024). Impaired autophagosome clearance and lysosomal degradation can result in the accumulation of autophagic substrates and incompletely degraded cellular components (Boland et al., 2008). In an Aβ25–35 model, increased levels of microtubule-associated protein 1 light chain 3-II (LC3-II), sequestosome 1 (p62), lysosome-associated membrane protein 2 (LAMP2), and cathepsin D (CatD), together with impaired autolysosome formation, indicated disrupted autophagic flux (Lee et al., 2023). Lee et al. further showed that Dex improved autophagic flux by promoting autophagosome–lysosome fusion and lysosomal degradation, thereby reducing the pathological accumulation of LC3-II and p62 (Lee et al., 2023). These findings suggest that Dex may restore the autophagy–lysosomal degradation axis and neuronal proteostasis. However, evidence regarding the regulation of autophagic flux by Dex remains limited. The specific molecular targets through which Dex regulates different stages of autophagy, including autophagosome formation and lysosomal maturation, as well as its interactions with upstream signaling pathways, require further investigation.

### 3.8 Regulation of synapse-associated proteins and synaptic plasticity

Postsynaptic density protein 95 (PSD-95) is a core scaffolding protein in the postsynaptic membrane that plays a crucial role in maintaining synaptic structural stability and functional integrity. It recruits glutamate receptors, such as N-methyl-D-aspartate receptors and α-amino-3-hydroxy-5-methyl-4-isoxazolepropionic acid receptors, as well as downstream signaling molecules such as CaMKII, thereby regulating synaptic signal transmission and plasticity (Taft and Turrigiano, 2014). CaMKII is activated by Ca^2+^/calmodulin binding and can undergo autophosphorylation at Thr286, which confers sustained kinase activity (Shioda and Fukunaga, 2017). In an Aβ-induced mouse model, reduced hippocampal PSD-95 expression and increased p-CaMKII levels were associated with cognitive dysfunction (Thapak et al., 2022). Lee et al. (2023) further demonstrated that Dex reversed Aβ-induced reductions in PSD-95 and elevations in p-CaMKII, thereby attenuating synaptic structural abnormalities. The imbalance between reduced PSD-95 expression and excessive CaMKII phosphorylation may contribute to synaptic dysfunction and cognitive impairment in AD-related models (Thapak et al., 2022). Dex may restore synaptic structural integrity by reversing these protein abnormalities (Lee et al., 2023). No obvious toxicity was observed under the specific *in vivo* and organotypic hippocampal slice culture conditions examined (Lee et al., 2023); however, these findings should not be interpreted as evidence of long-term clinical safety. Current evidence mainly focuses on postsynaptic markers. Whether Dex regulates presynaptic function, neurotransmitter release, or synaptic pruning remains unclear. Future studies integrating electrophysiological recordings with synaptosomal proteomic analyses may help clarify whether Dex modulates presynaptic function, neurotransmitter release, and synaptic remodeling in AD.

### 3.9 Integrated signaling network and mechanistic crosstalk

Rather than acting on isolated pathological processes, Dex appears to influence an interconnected network involving neuroinflammation, oxidative stress, mitochondrial injury, proteostasis, and neuronal survival. α₂-AR-dependent suppression of NF-κB signaling may attenuate inflammatory mediator production (Li et al., 2020; Luo et al., 2022). In 5 × FAD mice, Dex treatment was also associated with reduced BACE1 expression and amyloidogenic processing (Luo et al., 2022). Reduced inflammatory activity may, in turn, limit ROS generation and secondary mitochondrial injury (Ma et al., 2024; Hu et al., 2022; Qiao et al., 2023; Chen et al., 2021). Conversely, mitochondrial dysfunction and oxidative stress may further amplify inflammasome activation and apoptotic signaling, thereby contributing to a self-reinforcing pathological cycle (Xu et al., 2025; Guo et al., 2026; Liang et al., 2022; Chen et al., 2021). Restoration of autophagic flux may provide an additional mechanism for maintaining neuronal proteostasis (Lee et al., 2023; Nixon and Rubinsztein, 2024; Boland et al., 2008). Recent evidence also links α₂-AR-dependent suppression of ROS to XIAP/MDM2/p53 signaling, providing a mechanistic bridge between oxidative stress and neuronal apoptosis (Wu et al., 2026). In parallel, modulation of mTOR-related ferroptotic signaling and BDNF-associated neurotrophic pathways may contribute to neuronal survival and synaptic stability (Qiao et al., 2023; Wang et al., 2019). Although PI3K/Akt signaling has been implicated in AD-related oxidative-stress regulation (Lin et al., 2022), direct evidence for Dex-mediated PI3K/Akt signaling in AD models remains insufficient. Similarly, direct involvement of Nrf2/ARE signaling in Dex-mediated neuroprotection in chronic AD remains to be established. These interconnected mechanisms and the relative strength and limitations of the supporting evidence are summarized in Figure 1.

**Figure 1:**
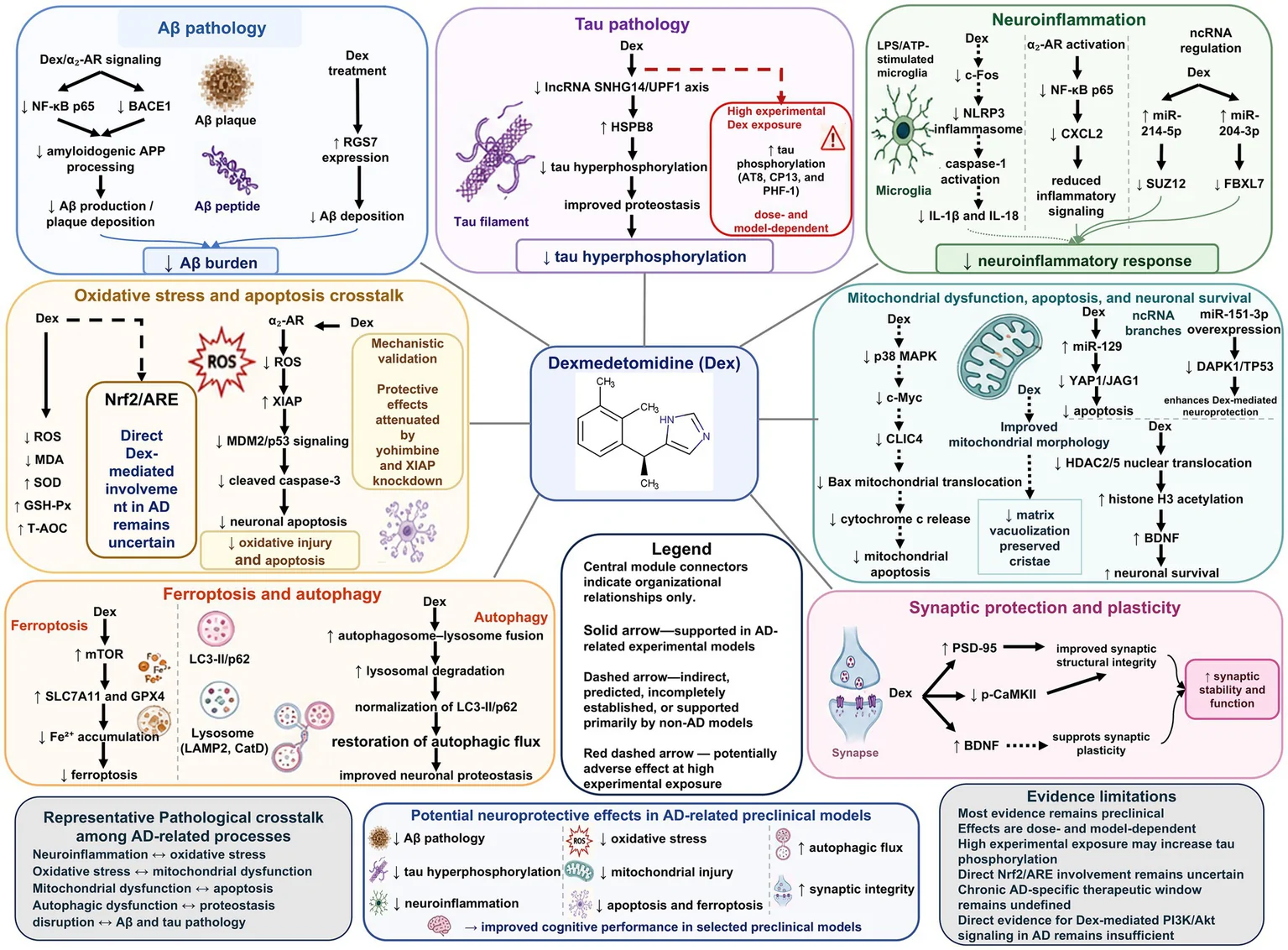
Integrated molecular mechanisms and potential neuroprotective actions of dexmedetomidine in Alzheimer’s disease. The schematic integrates reported Dex-associated pathways involving amyloid metabolism, tau regulation, neuroinflammation, oxidative stress, mitochondrial homeostasis, cell death pathways, autophagy, and synaptic regulation.

## 4 Translational considerations, dosing, pharmacokinetics, and safety

Despite encouraging preclinical findings, several barriers currently limit the direct translation of Dex into an AD-directed therapy. First, the available evidence is dominated by cellular and animal studies employing markedly different AD models, treatment schedules, administration routes, and exposure durations. As summarized in Table 1, the available preclinical studies used widely varying Dex doses and concentrations across different AD-related models. Neuroprotective effects have been reported at 4 μg/kg/day for 4 weeks in 5 × FAD mice (Wu et al., 2026), at 40–80 μg/kg in an Aβ25–35-induced mouse model (Lee et al., 2023), and at 100 μg/kg in 5 × FAD mice (Luo et al., 2022), whereas 300 μg/kg increased tau phosphorylation under specific experimental conditions (Whittington et al., 2015). *In vitro*, Wu et al. selected 10 μM Dex for subsequent mechanistic experiments because it provided the strongest protection among the tested concentrations, while noting that this concentration exceeded clinically relevant plasma levels (Wu et al., 2026). These heterogeneous experimental exposures cannot be directly interpreted as an optimal human dose and instead underscore the need for systematic pharmacokinetic/pharmacodynamic evaluation.

Second, the established clinical pharmacology of Dex must be considered when evaluating its potential chronic use in older individuals with AD. In healthy adults, Dex has an elimination half-life of approximately 2.1–3.1 h and a clearance of approximately 0.6–0.7 L/min, and it undergoes extensive hepatic metabolism (Weerink et al., 2017). Dex is primarily used clinically as a sedative and can produce dose-dependent sedation and cardiovascular effects, particularly hypotension and bradycardia (Weerink et al., 2017). These effects may be especially relevant in older patients with autonomic dysfunction, cardiovascular comorbidities, polypharmacy, or an increased risk of falls. Consequently, experimental neuroprotective activity does not necessarily imply an acceptable benefit–risk profile for chronic administration in patients with AD.

The available clinical evidence should also be interpreted cautiously because disease-modifying efficacy in AD remains unproven. A recruiting phase 2 trial (NCT06052254) is currently evaluating a dexmedetomidine transdermal system for agitation associated with dementia of the Alzheimer’s type; importantly, the primary therapeutic target is agitation rather than Aβ or tau pathology (ClinicalTrials.gov, 2026). Therefore, such studies cannot establish disease-modifying efficacy. Future studies should evaluate clinically feasible exposure regimens, pharmacokinetic/pharmacodynamic relationships, target-engagement biomarkers, AD-relevant cognitive and pathological outcomes, and cardiovascular safety.

## 5 Conclusions and perspectives

Current preclinical evidence suggests that Dex, a highly selective α₂-AR agonist, may modulate several AD-related pathological processes through multiple mechanisms. In Aβ-induced models, Dex has been reported to reverse reductions in PSD-95 and elevations in p-CaMKII, suggesting a potential role in preserving synaptic integrity (Lee et al., 2023). Dex may also influence Aβ pathology through α₂-AR-dependent signaling, BACE1 regulation, and RGS7-associated mechanisms (Luo et al., 2022; Yang et al., 2025; Nazli et al., 2026). Recent evidence further suggests that α₂-AR-dependent modulation of the ROS/XIAP/MDM2/p53 axis may link oxidative stress to neuronal apoptosis (Wu et al., 2026).

Despite encouraging preclinical findings, the translational evidence for Dex in AD remains preliminary because available studies vary substantially in experimental model, dose, route of administration, and treatment duration, and AD-specific clinical efficacy has not yet been established. First, further studies are needed to elucidate the molecular network through which Dex regulates AD pathology, with particular emphasis on its dose- and context-dependent effects on tau phosphorylation and its interactions with neuroinflammatory, oxidative, mitochondrial, autophagic, and synaptic pathways. Second, most available studies have focused on specific animal models of AD. Future investigations should systematically evaluate the efficacy of Dex across different stages of pathological progression, such as early preventive intervention and late therapeutic intervention, and in models with distinct genetic backgrounds, such as APP/PS1 and 5 × FAD mice, to clarify its specificity and cross-model generalizability. Finally, from a translational perspective, based on the currently identified targets of Dex, future studies may explore structural modification and pharmacodynamic optimization of Dex or systematically assess combination strategies with other active molecules that exert complementary neuroprotective effects.

More broadly, recent studies of other pleiotropic neuroprotective candidates, such as fisetin and anatabine, have highlighted the potential value of simultaneously targeting inflammatory, oxidative, and proteostatic pathways in neurodegenerative disorders (Singh et al., 2026a; Singh et al., 2026b). These findings provide conceptual support for network-based therapeutic strategies, although the efficacy and safety of each candidate, including Dex, require independent validation.

## Glossary

AD: Alzheimer’s disease
Aβ: amyloid-β
α₂-AR: α₂-adrenergic receptor
ApoE4: apolipoprotein E4
APP: amyloid precursor protein
ARE: antioxidant response element
BACE1: β-site amyloid precursor protein-cleaving enzyme 1
Bax: Bcl-2-associated X protein
Bcl-2: B-cell lymphoma 2
BDNF: brain-derived neurotrophic factor
CaMKII: calcium/calmodulin-dependent protein kinase II
CatD: cathepsin D
CLIC4: chloride intracellular channel 4
CXCL2: C-X-C motif chemokine ligand 2
DAMPs: damage-associated molecular patterns
DAPK1: death-associated protein kinase 1
Dex: dexmedetomidine
ERK: extracellular signal-regulated kinase
FBXL7: F-box and leucine-rich repeat protein 7
GPX4: glutathione peroxidase 4
GSH-Px: glutathione peroxidase
GSK-3β: glycogen synthase kinase-3β
HDAC2/5: histone deacetylases 2 and 5
HSPB8: heat shock protein family B member 8
i.c.v.: intracerebroventricular
i.p.: intraperitoneal
JAG1: Jagged1
IL-1β: interleukin-1β
IL-18: interleukin-18
LAMP2: lysosome-associated membrane protein 2
LC3-II: microtubule-associated protein 1 light chain 3-II
lncRNA: long non-coding RNA
LPS: lipopolysaccharide
MAPK: mitogen-activated protein kinase
MDA: malondialdehyde
MDM2: mouse double minute 2 homolog
miR: microRNA
mTOR: mechanistic target of rapamycin
ncRNA: non-coding RNA
NF-κB: nuclear factor kappa B
NFTs: neurofibrillary tangles
NLRP3: NOD-like receptor family pyrin domain-containing 3
Nrf2: nuclear factor erythroid 2-related factor 2
OHSC: organotypic hippocampal slice culture
PC12: pheochromocytoma 12 cell line
p-CaMKII: phosphorylated calcium/calmodulin-dependent protein kinase II
PP2A: protein phosphatase 2A
PSD-95: postsynaptic density protein 95
p-Tau: phosphorylated tau
RGS7: regulator of G-protein signaling 7
ROS: reactive oxygen species
SH-SY5Y: human neuroblastoma cell line
SLC7A11: solute carrier family 7 member 11
SNHG14: small nucleolar RNA host gene 14
SOD: superoxide dismutase
SUZ12: suppressor of zeste 12 homolog
T-AOC: total antioxidant capacity
TP53: tumor protein p53
UPF1: UPF1 RNA helicase
XIAP: X-linked inhibitor of apoptosis protein
YAP1: Yes-associated protein 1
5 × FAD: five familial Alzheimer’s disease mutations
